# Quadriceps recovery and pain relief in knee osteoarthritis rats by cog polydioxanone filament insertion

**DOI:** 10.1093/rb/rbae077

**Published:** 2024-06-21

**Authors:** Myeounghoon Cha, Heyji Bak, Sun Joon Bai, Bae Hwan Lee, Jun Ho Jang

**Affiliations:** Department of Physiology, Yonsei University College of Medicine, Seoul 03722, Republic of Korea; Department of Physiology, Yonsei University College of Medicine, Seoul 03722, Republic of Korea; Department of Anesthesiology and Pain Medicine, Anesthesia and Pain Research Institute, Yonsei University College of Medicine, Seoul 03722, Republic of Korea; Department of Physiology, Yonsei University College of Medicine, Seoul 03722, Republic of Korea; Brain Korea 21 PLUS Project for Medical Science, Yonsei University College of Medicine, Seoul 03722, Republic of Korea; Brain Research Institute, Yonsei University College of Medicine, Seoul 03722, Republic of Korea; R&D Center, OV MEDI Co., Ltd, Gunpo 15847, Republic of Korea

**Keywords:** muscle restoration, osteoarthritis, pain, knee, quadriceps, satellite cell, macrophage

## Abstract

Quadriceps muscles play a pivotal role in knee osteoarthritis (OA) progression and symptom manifestation, particularly pain. This research investigates the therapeutic effectiveness of muscle enhancement and support therapy (MEST), a recently developed device intended for intramuscular insertion of cog polydioxanone filaments, in quadriceps restoration to alleviate OA pain. Knee OA was induced in Sprague Dawley rats via monoiodoacetate injections. MEST or sham treatment was performed in OA or Naive rat quadriceps. Pain was assessed using paw withdrawal threshold and weight bearing. Quadriceps injury and recovery via MEST were evaluated using biomarkers, tissue morphology, muscle mass, contractile force and hindlimb torque. Satellite cell and macrophage activation, along with their activators, were also assessed. Data were compared at 1- and 3-weeks post-MEST treatment (M-W1 and M-W3). MEST treatment in OA rats caused muscle injury, indicated by elevated serum aspartate transferase and creatinine kinase levels, and local β-actin changes at M-W1. This injury triggered pro-inflammatory macrophage and satellite cell activation, accompanied by heightened interleukin-6 and insulin-like growth factor-1 levels. However, by M-W3, these processes gradually shifted toward inflammation resolution and muscle restoration. This was seen in anti-inflammatory macrophage phenotypes, sustained satellite cell activation and injury markers regressing to baseline. Quadriceps recovery in mass and strength from atrophy correlated with substantial OA pain reduction at M-W3. This study suggests that MEST-induced minor muscle injury triggers macrophage and satellite cell activation, leading to recovery of atrophied quadriceps and pain relief in OA rats.

## Graphical Abstract

**Figure:**
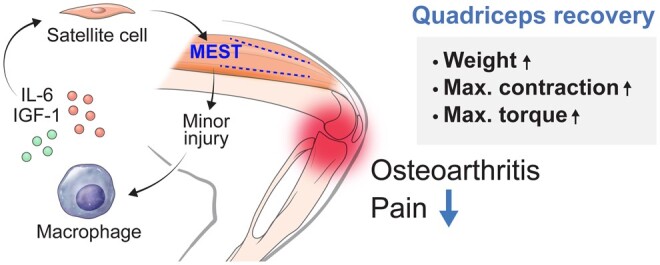

## Introduction

Osteoarthritis (OA), also known as degenerative joint disease, is a common joint disorder that results from the progressive loss of articular cartilage [1, 2], impacting millions worldwide [3]. This loss of cartilage can occur due to a variety of factors, including wear and tear, joint injury, and genetic predisposition [1, 2]. Despite potentially promising approaches targeting OA joints with drugs [4, 5], stem cells [6, 7] and biomaterials [8–10], current treatment options primarily focus on alleviating symptoms, especially pain [11], as there are no proven disease-modifying treatments for OA at present.

Previous studies have shown the significant roles of associated skeletal muscles in the development of OA as well as its symptoms and function [12–17]. For instance, weakness in the quadriceps muscles is a contributing factor to the development of knee OA [17]. Moreover, a randomized clinical trial unveiled evidence that quadricep strengthening exercises yields favorable outcomes by alleviating pain, enhancing functionality and elevating the overall quality of life for knee OA patients [16]. Building on these studies, a new therapeutic strategy targeting weakened quadriceps has been recently introduced for OA-induced pain management in knee OA rats [18, 19]. In those studies, a new muscle enhancement and support therapy (MEST) device was utilized to intramuscularly deliver biodegradable cog polydioxanone (PDO) filaments, designed to anchor and stimulate the surrounding muscle tissues within the quadriceps. The outcome showed effective relief of peripheral sensitization and pain, alongside quadriceps recovery and a local increase in muscle restoration factors. Importantly, the safety and efficacy of MEST for the management of knee OA pain have also been established in a randomized blinded clinical trial [20]. While mechanisms related to muscle stimulation and support have been proposed [18, 19], the precise mechanisms underlying MEST treatment remain largely unknown.

Considering the potential of cog PDO filaments to induce minor injuries to the surrounding quadriceps, this study aimed to elucidate one of the possible mechanisms through which such injuries contribute to the restoration of atrophied quadriceps and relief from OA pain. We examined the roles of satellite cells [21, 22] and macrophages [23, 24] as pivotal contributors to muscle regeneration post-injury. The MEST-induced muscle injury may trigger an inflammatory response, activating satellite cells to regenerate muscle tissue beyond its pre-injury state, ultimately contributing to the relief from OA pain. Similar injury and over-recovery mechanisms have been proposed in muscle hypertrophy due to exercise-induced muscle damage [25]. In addition, the strategy of deliberate irritation and inflammation to promote tissue repair and strengthening is widely utilized in the management of musculoskeletal pain [26, 27].

The current study examined the efficacy of MEST treatment for alleviating pain in a monoiodoacetate (MIA)-induced OA animal model. Assessment of muscle injury and recovery following MEST involved analyzing biomarkers, tissue morphology, muscle mass and strength. Additionally, we investigated the activation of satellite cells, macrophages and associated activators. Early (1 week) and late (3 weeks) time points after MEST treatment (M-W1 and M-W3, respectively) were compared to track injury progression, muscle restoration and pain changes. Our findings suggest that MEST treatment, coupled with a minor injury and subsequent recovery of atrophic quadriceps in knee OA rats, contributes to pain relief.

## Materials and methods

### Animals

The experimental protocols received approval from the Institutional Animal Care and Use Committee of Yonsei University (#2021-0285), and all procedures were carried out in compliance with relevant regulations and guidelines. Reporting of the experiments adheres to the Animal Research: Reporting of *In Vivo* Experiments (ARRIVE) guidelines. Sprague Dawley rats (Orient Bio, Seongnam, Korea; 6–7 weeks of age at the start of experiments; both sexes) that were housed in groups of three to four, with unrestricted access to food and water under a 12-h light/dark cycle, were used in this study.

### OA induction

We employed the MIA model of OA, a well-established and widely used chemical model, most suitable for studying the effectiveness of new treatments in pain associated with OA [28]. Rats received intra-articular injections of MIA (Sigma Aldrich, Milwaukee, WI, USA) under brief anesthesia at weeks 0 and 2, with a first dose of 1 mg and a second dose of 3 mg, each in 30 μl of saline. A second MIA injection was included in this study as in our prior study, in which some behavioral deficits in OA animals were not evident 2 weeks after the first MIA injection [18].

### MEST treatment

The details of MEST and its treatment technique were described in a previous study [18]. In brief, a new medical device, MEST (MEST-B2375, OV MEDI, Seoul, Korea), was employed for the intramuscular insertion of a specifically designed PDO filament. This apparatus consists of a protective cap, a 23-gauge needle, a PDO filament (outer diameter 0.3 mm) featuring bi-directional cogs, and a catheter that safeguards the cogs upon insertion in the opposite direction. Under brief anesthesia, a shaved and incised area was created over the left hindlimb thigh muscles. The MEST needle, carrying a folded PDO filament, was inserted to approximately 1/3 of the total thigh muscle length from the knee, then advanced towards the knee itself. Upon reaching the distal femur area, the needle and catheter were sequentially withdrawn, leaving the PDO filament securely attached to the quadriceps muscles through its cogs. A total of five filaments were evenly administered across the lateral, medial and ventral regions of the thigh muscles. Any exposed PDO filament extending beyond the muscle was excised using scissors, and the incision was closed. Sham treatment was conducted similarly, excluding the insertion of the PDO filament.

### Experimental design

A total of 138 animals were randomly assigned to groups based on treatments and experimental periods (up to M-W1 or M-W3) using a simple randomization approach without control for potential confounders. The M-W1 and M-W3 time points were chosen to monitor the progression of changes in affected muscles and pain levels. This decision was based on the observation that by week 5 following MEST treatment, both pain relief and muscle restoration had matured without indications of muscle inflammation [18].

All experiments were conducted under blinded conditions with respect to the following assigned groups: (i) Naive: no treatment (*n* = 12 for both M-W1 and M-W3 time periods); (ii) MIA: rats were subjected to first and second MIA injections at weeks 0 and 2 (*n* = 11 for M-W1, *n* = 12 for M-W3 time periods); (iii) MIA+MEST: same as the MIA group, with MEST treatment at week 3 (*n* = 12 for both M-W1 and M-W3 time periods); (iv) MIA+Sham: same as the MIA group, with sham needle treatment at week 3 (*n* = 12 for M-W1, *n* = 10 for M-W3 time periods); (v) Naive+MEST: same as the Naive group, with MEST treatment at week 3 (*n* = 12 for M-W1, *n* = 11 for M-W3 time periods); and (vi) Naive+Sham: same as the Naive group, with sham needle treatment at week 3 (*n* = 11 for both M-W1 and M-W3 time periods). The sample sizes were determined based on a previous study that used the same animal model and MEST treatment for similar experimental procedures [18].

No animals were excluded during the behavioral experiments. After that, half of them were assigned to western blot experiments (*n* = 4–6), while the other half were assigned to muscle contraction tests followed by blood sample collection (*n* = 3–6) and then immunohistochemistry staining (*n* = 4). Animals not included in the data analysis were used to set the experimental conditions. Additionally, due to the variability of serum biomarkers data, we added additional available animals solely for serum biomarker analysis not included in other experiments, bringing the total to *n* = 5–12.

### von Frey test

Mechanical stimuli were delivered to the plantar surface of the left hind paw using an electronic von Frey apparatus (no. 38450; UGO Basile, Varese, Italy). A blunt probe (0.5 mm in diameter) was applied with increasing force until the rat withdrew its paw, and the threshold force required to elicit a paw withdrawal response was automatically recorded by the apparatus as the paw withdrawal threshold (PWT). Five values were recorded and averaged to obtain the final PWT measurement for each rat.

### Weight-bearing test

The rats were positioned in a plexiglass holder (10 cm in diameter, 30 cm high) in such a way that each hind paw was placed on a separate force plate of an incapacitance tester (Linton Instrumentation, Norfolk, UK). The force exerted by each hindlimb was measured and averaged over a 5-s period. Each data point was obtained by taking the mean of three 5-s readings. To calculate the change in hind paw weight distribution, the weight-bearing ratio (WBR) of the left over the right hindlimb was determined.

### Grip strength test

The grip strength meter (DS2-50N, Columbus Instruments, Columbus, OH, USA) consisted of a T-shaped metal bar connected to a force transducer. The experimenter then held the rats gently by the base of the tail, allowing them to grasp the metal bar with their hindpaws. The rats were pulled backwards by the tail until grip was lost, and the peak force of each measurement was automatically recorded by the device. For each hindpaw grip strength measure, three measurements were taken, and the average value was used.

### Muscle contractility measurement, *in vivo*

Hindlimb torque resulting from quadriceps contraction was assessed under anesthesia (urethane, 0.5 g/kg, i.p.). To measure hindlimb torque accurately, the knee joint was fixed in an axial position and a digital torque meter (HP-100, HIOS, Osaka, Japan) was employed. Quadriceps contraction was induced through electrical stimulation targeting the femoral nerve using needle electrodes. The precise electrode placement was determined by observing a series of isometric twitches and isolated knee extensions.

Following torque measurement, the quadriceps tendon connected to the knee was released and attached to a force transducer (MLTF050/ST, AD Instruments, Colorado Springs, CO, USA) to directly measure muscle tension. Three measurements of hindlimb torque and muscle tension were taken, at approximately 1-min intervals, and the average was calculated.

### Muscle weight measurement

After contractility measurements, the whole quadriceps muscle was quickly extracted and frozen on dry ice. A 3-mm thick slice of muscle tissue taken 5 mm from the distal end of the quadriceps was used for weight measurement.

### Blood sample collection

Venous blood (3 ml) was collected using a syringe. The collected blood was transferred to a serum separation tube, left to clot, and subsequently centrifuged at 2000 rpm for 10 min. The resulting serum was stored in a deep freezer at −80°C until it was subjected to biochemical analysis. Concentrations of aspartate transferase (AST), lactate dehydrogenase (LDH) and creatine kinase (CK) were determined using a spectrophotometer analyzer.

### Tissue staining

The rats under anesthesia were transcardially perfused with normal saline (0.9% NaCl) followed by 4% paraformaldehyde in 0.1 M sodium phosphate buffer (pH 7.4). The quadriceps muscle was postfixed by soaking in 30% sucrose in phosphate-buffered saline (PBS) and cryosectioned to a thickness of 30 μm using a cryostat (HM525, Thermo Scientific, Waltham, MA, USA).

For hematoxylin and eosin staining, staining was carried out using alcoholic eosin, and dehydration was performed using a series of ethanol solutions (70%, 90% and 100%). The slides were cleared in xylene, mounted and coverslipped.

For immunohistochemistry, the sections were incubated overnight at 4°C with primary antibodies and rinsed with PBS containing 0.3% Tween-20. Subsequently, a mixture of secondary antibodies was applied and incubated for 2 h at room temperature. All sections were mounted with 4',6-diamidino-2-phenylindole (DAPI, Vector Laboratories, Burlingame, CA, USA). Image acquisition was performed using a laser scanning confocal microscope (LSM 700; Carl Zeiss, Jena, Germany). Four images were acquired from each animal. The antibodies used are listed in Supplementary Table S1.

### Western blot

The samples were homogenized and centrifuged at 15 000 rpm for 10 min. Protein samples were denatured, separated and transferred onto membranes (Merck Millipore, Darmstadt, Germany). The lower and upper portions of each membrane were cut in accordance with the ladder guide after protein transfer and before exposure to avoid potential signal interference. The intensity of the protein bands was quantified using the LAS system (Amersham ImageQuant 800, Washington DC, USA). At least three independent western blots were quantified for data analysis. The antibodies used are listed in Supplementary Table S1.

### Data analysis

There was no data that had been excluded. All data were presented as mean ± standard error of the mean (SEM). Naive group was used as a reference for all normalized data. The differences among groups measured once at a specific time point were analyzed using one-way analysis of variance (ANOVA) followed by Tukey’s multiple comparison test (four groups) or unpaired *t*-test (two groups). For group differences measured repeatedly at multiple time points or at a specific time point with a different experimental condition, two-way repeated measures ANOVA followed by Tukey’s (four groups) or Sidak’s (two groups) test was used. Statistical significance was set at *P* < 0.05. All statistical analyses were performed using the Prism 7.0 (GraphPad Software, Boston, USA) software.

## Results

### Pain relief by MEST

**Figure 1.**
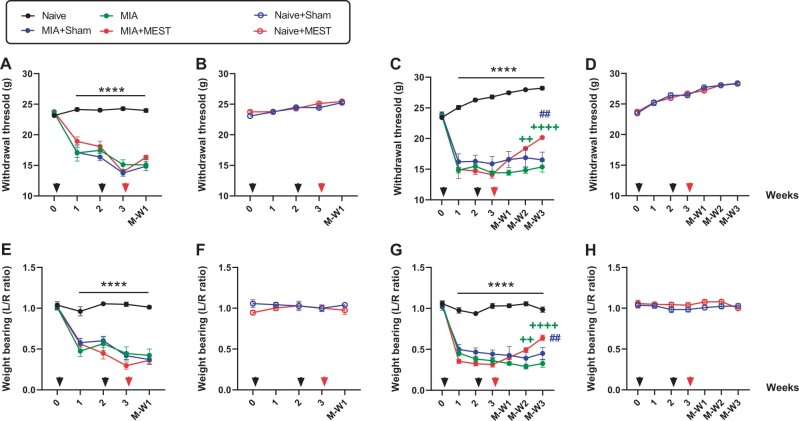
MEST relieves MIA-induced pain. PWT to von Frey filaments up to M-W1 (**A** and **B**) or M-W3 (**C** and **D**). WBR of the left over right hindlimb up to M-W1 (**E** and **F**) or M-W3 (**G** and **H**). Black arrowheads indicate MIA injections and red arrowhead indicates MEST treatment. **** *P* < 0.0001 MIA, MIA+Sham or MIA+MEST vs. Naive. ++ *P* < 0.01, ++++ *P* < 0.0001 vs. MIA. ## *P* < 0.01 vs. MIA+Sham. *n* = 10–12 per group. Data are presented as mean ± SEM. Data were analyzed using two-way repeated measures ANOVA followed by Tukey’s (A, C, E and G) or Sidak’s (B, D, F and H) test.

First, we examined the effect of MEST on MIA-induced pain (Figure 1A–H). Following MIA injection, the animals exhibited a reduction in PWT within 1 week of the first injection, which persisted until M-W3 compared to the Naive group (Figure 1A and C). Although MEST treatment at week 3 did not reverse the decreased PWT early at M-W1 (Figure 1A), MEST gradually and significantly ameliorated it at M-W2 and M-W3 compared to the MIA or MIA+Sham groups (Figure 1C). MEST treatment showed no impact on PWT reduction in Naive animals (Figure 1B and D). Additionally, sham treatment did not alter PWT in either MIA or Naive animals (Figure 1A–D).

WBR remained relatively stable in Naive animals throughout the study, whereas MIA-injected animals displayed a noticeable decrease in WBR within a week, which persisted until M-W3 (Figure 1E and G). However, MEST treatment at week 3 efficiently mitigated the MIA-induced WBR reduction. Significant reverses were evident at M-W2 and M-W3 (Figure 1G), although not at M-W1 (Figure 1E), compared to the MIA or MIA+Sham groups. MEST treatment had no significant impact on WBR among Naive animals (Figure 1F and H), and sham treatment did not influence WBR in either the MIA or Naive animals (Figure 1E–H).

Grip strength in the hindpaws showed no significant differences among groups throughout the 4-week study period (Supplementary Figure S1). This finding suggests that hindpaw grip strength was not primarily influenced by knee pain or quadriceps condition. As a result, further experiments investigating hindpaw grip strength were not pursued.

### Muscle injury by MEST

**Figure 2.**
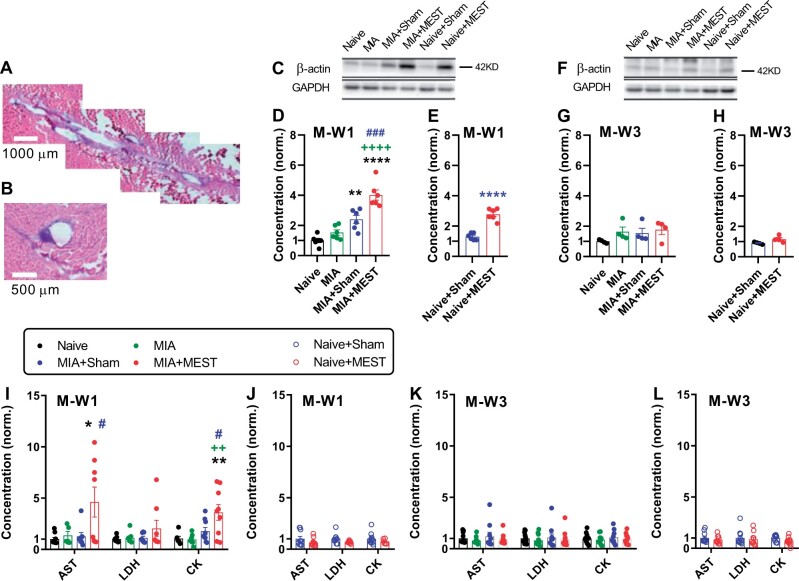
MEST induces minor muscle injury. (**A** and **B**) Examples of longitudinal (A) and cross-sectional (B) traces of quadriceps injury caused by an inserted filament, observed at M-W3 in the MIA+MEST group. (**C**–**H**) Western blot analysis of β-actin levels in quadriceps. Representative western blots are shown in (C) and (F). Uncropped original bands are shown in Supplementary Figure S3. β-actin levels at M-W1 (D and E) and M-W3 (G and H) were normalized. *n* = 4–6 per group. (**I**–**L**) Normalized serum levels of AST, LDH and CK at M-W1 (I and J) and M-W3 (K and L) are shown. *n* = 5–12 per group. The Naive group served as the reference for normalization. * *P* < 0.05, ** *P* < 0.01, **** *P* < 0.0001 vs. Naive or Naive+Sham. ++ *P* < 0.01, ++++ *P* < 0.0001 vs. MIA. # *P* < 0.05, ### *P* < 0.001 vs. MIA+Sham. Data are presented as mean ± SEM. The data were analyzed using one-way ANOVA followed by Tukey’s multiple comparison test (D, G, I and K) or unpaired *t*-test (E, H, J and L).

Subsequently, we examined whether the MEST treatment triggers muscle injury (Figure 2A–L). Longitudinal (Figure 2A) and cross-sectional (Figure 2B) views of quadriceps tissue, shown as an example obtained from the MIA+MEST group at M-W3, revealed traces of mechanical damage by inserted MEST filament.

At M-W1, β-actin levels in quadriceps were significantly higher in the MIA+MEST group compared to the Naive, MIA or MIA+Sham groups, indicating the presence of local muscle injury due to MEST (Figure 2C and D). The Sham procedure also increased local β-actin levels in the MIA group, but to a lesser extent at M-W1 (Figure 2C and D). In Naive animals, MEST treatment, but not sham treatment, increased β-actin levels at M-W1 (Figure 2C–E). However, these changes observed among groups were no longer significant at M-W3 (Figure 2F–H).

Regarding serum biomarkers for muscle injury, such as AST and CK, their levels were higher in the MEST+MIA animals compared to the Naive, MIA or MIA+Sham groups at M-W1. Although the LDH level in the MIA+MEST group showed a tendency to increase, it did not reach statistical significance (Figure 2I). However, these differences were not observed among all groups at M-W3 (Figure 2K). In Naive animals, serum levels of AST, LDH and CK with sham or MEST treatment did not differ at both M-W1 and M-W3 (Figure 2J and L).

### Muscle recovery by MEST

**Figure 3.**
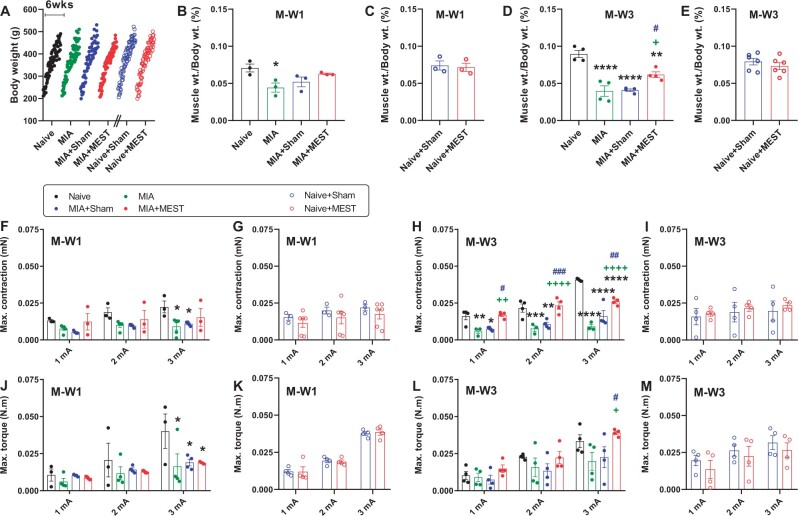
MEST restores atrophied quadriceps. (**A**–**E**) Recovery of muscle weight. (A) Consistent changes in whole body weight over 6 weeks among all groups. *n* = 10–12 per group. (B–E) The ratio of quadriceps slice weight to body weight was measured to assess muscle atrophy or recovery at M-W1 (B and C) and M-W3 (D and E). *n* = 3–6 per group. (**F**–**I**) Recovery of contraction force. The maximal contraction force and hindlimb torque force were measured to evaluate quadriceps contraction ability. The maximal contraction force of the quadriceps elicited by electrical stimulation at M-W1 (F and G) and M-W3 (H and I). *n* = 3–6 per group. (**J**–**M**) Recovery of torque force of the hindlimb. The maximal torque force of the hindlimb elicited by electrical stimulation of quadriceps at M-W1 (J and K) and M-W3 (L and M). *n* = 3–4 per group. Electrical stimulation with 1-ms pulse durations at amplitudes of 1, 2 or 3 mA was delivered to elicit quadriceps contraction and hindlimb torque. * *P* < 0.05, ** *P* < 0.01, *** *P* < 0.001, **** *P* < 0.0001 vs. Naive. + *P* < 0.05, ++ *P* < 0.01, ++++ *P* < 0.0001 vs. MIA. # *P* < 0.05, ## *P* < 0.01, ### *P* < 0.001 vs. MIA+Sham. Data are presented as mean ± SEM. Data were analyzed using one-way ANOVA followed by Tukey’s multiple comparison test (B and D) or unpaired *t*-test (C and E), and two-way repeated measures ANOVA followed by Tukey’s (F, H, J and L) or Sidak’s (G, I, K and M) test.

Muscle weakness or restoration was evaluated by measuring the muscle mass, muscle contraction strength and hindlimb torque (Figure 3A–M).

#### Muscle weight

Throughout the 6-week experimental period, the body weight of the animals remained consistent across all groups (Figure 3A). However, at M-W1, the ratio of muscle slice weight to body weight was lower in MIA animals compared to the Naive group (Figure 3B). The ratio in sham- or MEST-treated Naive animals was similar to that of the Naive animals (Figures 3B and C). At M-W3, all MIA, MIA+Sham and MIA+MEST animals exhibited a decreased muscle weight ratio to body weight compared to Naive animals. However, the MIA+MEST group displayed a higher ratio than both the MIA and MIA+Sham animals (Figure 3D), indicating successful restoration of quadriceps weight in the MEST-treated group. The ratios between sham- and MEST-treated Naive animals were not different (Figure 3E).

#### Muscle contraction

The force of maximal contraction in the quadriceps muscle, elicited by electrical stimulation with varying amplitudes (1, 2 or 3 mA) and 1-ms pulse duration, exhibited a decrease in MIA or MIA+Sham animals at M-W1 compared to the Naive group (Figure 3F). In contrast, sham or MEST treatment in Naive animals did not impact the contraction force (Figure 3G). By M-W3, the MIA and MIA+Sham groups displayed reduced contraction force compared to Naive animals, while the MIA+MEST group demonstrated a significantly higher contraction force than both the MIA and MIA+Sham groups, indicating successful restoration of quadriceps strength (Figure 3H). There was no significant difference in the contraction force between sham- and MEST-treated Naive animals (Figure 3I). Similar results were obtained when using 10-ms pulse duration electrical stimulation (Supplementary Figure S2A–D).

#### Hindlimb torque

At M-W1, a notable decrease in the maximal force of hindlimb torque, produced by electrical stimulation with varying amplitudes (1, 2 or 3 mA) and 1-ms pulse duration, was observed in the MIA, MIA+Sham and MIA+MEST groups compared to the Naive group (Figure 3J). However, sham or MEST treatment in Naive animals did not result in significant changes in torque force (Figure 3K). Similar patterns were observed at M-W3, with the MIA+MEST group showing higher torque force compared to both the MIA and MIA+Sham groups (Figure 3L), indicating successful recovery of quadriceps strength in the MEST-treated group. There was no significant difference in the maximal torque force between sham- and MEST-treated Naive animals (Figure 3M). Additionally, similar results were obtained when using 10-ms pulse duration electrical stimulation (Supplementary Figure S2E–H).

### Macrophage activation by MEST

**Figure 4.**
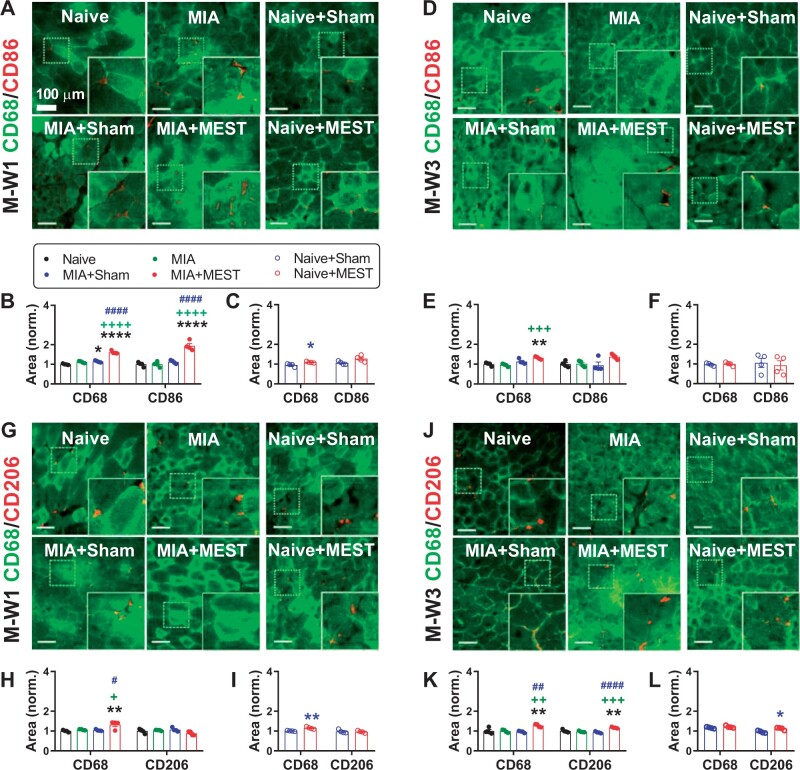
MEST activates macrophages. (**A**–**L**) Immunohistochemical analysis of macrophages. Representative images of immunostaining (A, D, G and J). Each inset indicated by a solid line represents a magnified view of the corresponding dotted area. Scale bar, 100 μm. The positive areas of CD86, an M1 pro-inflammatory macrophage marker (B, C, E and F), or CD206, an M2 anti-inflammatory macrophage marker (H, I, K and L), co-stained with CD68, a pan-macrophage marker, in quadriceps at M-W1 or M-W3, was normalized. The Naive group served as the reference for normalization. * *P* < 0.05, ** *P* < 0.01, **** *P* < 0.0001 vs. Naive or Naive+Sham. + *P* < 0.05, ++ *P* < 0.01, +++ *P* < 0.001, ++++ *P* < 0.0001 vs. MIA. # *P* < 0.05, ## *P* < 0.01, #### *P* < 0.0001 vs. MIA+Sham. *n* = 4 per group. Data are presented as mean ± SEM. Data were analyzed using one-way ANOVA followed by Tukey’s multiple comparison test (B, E, H and K) or unpaired *t*-test (C, F, I and L).

To delve into the activation of macrophages, we analyzed the expression of macrophage markers (Figure 4A–L). At M-W1, the quadriceps sections of MIA+MEST animals exhibited a significantly higher expression of Cluster of Differentiation (CD)68, a pan-macrophage marker, compared to the Naive, MIA and MIA+Sham groups (Figure 4A, B, G and H). Additionally, the expression of CD86, an M1 pro-inflammatory macrophage marker, was also elevated in the MIA+MEST group compared to the other groups at M-W1 (Figure 4A and B), indicating an inflammatory response induced by MEST in MIA animals. At M-W3, the CD68 expression remained elevated in the MIA+MEST group (Figure 4D, E, J and K). However, the expression of CD86 was no longer significantly different among groups at M-W3 (Figure 4D and E). Instead, the expression of CD206, an M2 anti-inflammatory macrophage marker, was higher in the MIA+MEST group compared to the Naive, MIA and MIA+Sham groups at M-W3 (Figure 4J and K), while no significant difference was observed at M-W1 (Figure 4G and H). Furthermore, MEST treatment in Naive animals resulted in a mild increase in CD68 expression at M-W1 and CD206 expression at M-W3 compared to the Naive+Sham group (Figure 4C, F, I and L).

### Satellite cell activation by MEST

**Figure 5.**
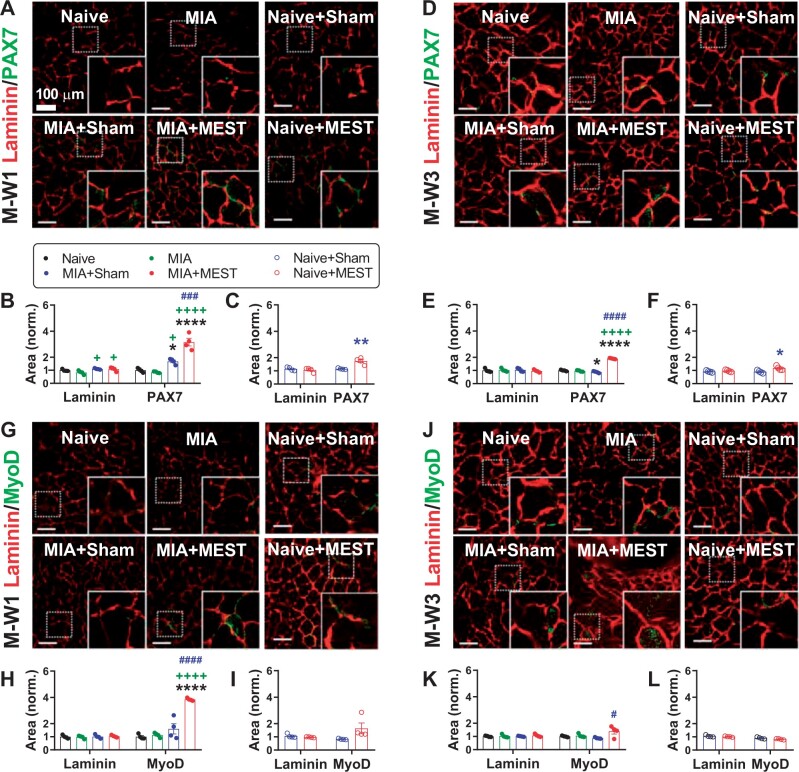
MEST activates satellite cells. (**A**–**L**) Immunohistochemical analysis of satellite cells. Representative images of immunostaining (A, D, G and J). Each inset indicated by a solid line represents a magnified view of the corresponding dotted area. Scale bar, 100 μm. The positive area of PAX7, a marker for satellite cells (B, C, E and F) or MyoD, a satellite cell activation marker (H, I, K and L), co-stained with laminin, in quadriceps at M-W1 or M-W3, was normalized. The Naive group served as the reference for normalization. * *P* < 0.05, ** *P* < 0.01, **** *P* < 0.0001 vs. Naive or Naive+Sham. + *P* < 0.05, ++++ *P* < 0.0001 vs. MIA. # *P* < 0.05, ### *P* < 0.001, #### *P* < 0.0001 vs. MIA+Sham. *n* = 4 per group. Data are presented as mean ± SEM. Data were analyzed using one-way ANOVA followed by Tukey’s multiple comparison test (B, E, H and K) or unpaired *t*-test (C, F, I and L).

We then shifted our focus to explore the activation of satellite cells by analyzing the expression of satellite cell markers (Figure 5A–L). The expression of laminin in the quadriceps sections of MIA+MEST or MIA+Sham animals was higher compared to the MIA group at M-W1 (Figure 5A and B), but the observation was not consistent (Figure 5G and H). MEST or sham treatment in Naive animals did not affect laminin levels at M-W1 (Figure 5A, C, G and I). At M-W3, there were no significant differences in laminin expression among all groups (Figure 5D–F and J–L). The expression of PAX7, a marker for satellite cells, was significantly higher in the MIA+MEST animals compared to the Naive, MIA and MIA+Sham groups at M-W1 (Figure 5A and B). Similarly, the expression of MyoD, a satellite cell activation marker, was also higher in the MIA+MEST group compared to the other groups at M-W1 (Figure 5G and H), indicating the initiation of muscle recovery in response to MEST treatment in MIA animals. At M-W3, while both PAX7 and MyoD expressions remained elevated in the MIA+MEST group (Figure 5D, E, J and K), the magnitude of the response was reduced compared to M-W1. Additionally, MEST treatment in Naive animals resulted in higher expression of PAX7 at M-W1 and M-W3 compared to the Naive+Sham group (Figure 5A, C, D and F). However, there were no significant differences in the expression of MyoD between the Naive+MEST and Naive+Sham groups at both M-W1 and M-W3 (Figure 5G, I, J and L).

### Satellite cell activators

**Figure 6.**
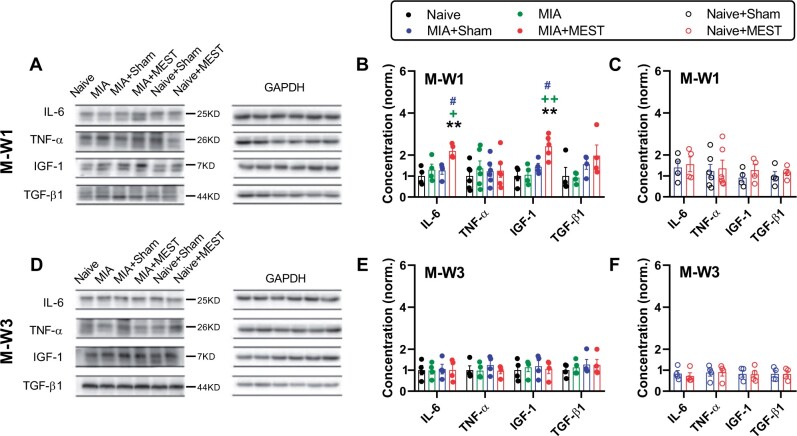
Changes in satellite cell activators. (**A**–**F**) Western blot analysis of IL-6, TNF-α, IGF-1 and TGF-β1 levels in quadriceps. Representative western blots are shown in (A) and (D). Uncropped original bands are shown in Supplementary Figure S3. The levels of protein/GAPDH control at M-W1 (B and C) and M-W3 (E and F) were normalized. The Naive group served as the reference for normalization. ** *P* < 0.01 vs. Naive. + *P* < 0.05, ++ *P* < 0.0 1 vs. MIA. # *P* < 0.05 vs. MIA+Sham. *n* = 4–6 per group. Data are presented as mean ± SEM. Data were analyzed using one-way ANOVA followed by Tukey’s multiple comparison test (B and E) or unpaired *t*-test (C and F).

We also examined the changes in inflammatory mediators and growth factors known to activate satellite cells (Figure 6A–F). At M-W1, the concentration of interleukin-6 (IL-6) and insulin-like growth factor-1 (IGF-1) in the quadriceps of MIA+MEST animals was significantly higher than those in the Naive, MIA and MIA+Sham groups (Figure 6A and B). However, this increase was not sustained at M-W3 (Figure 6D and E). In contrast, the levels of tumor necrosis factor alpha (TNF-α) and transforming growth factor beta-1 (TGF-β1) were not significantly different among all groups at both M-W1 and M-W3 (Figure 6A, B, D and E). The levels of IL-6, TNF-α, IGF-1 and TGF-β1 were not significantly different between sham- and MEST-treated Naive animals (Figure 6A, C, D and F).

## Discussion

Quadriceps weakness can worsen OA pain by destabilizing the knee joint, altering its biomechanics and promoting misalignment [29–32]. This weakness increases stress on the joint during weight-bearing activities, leading to cartilage breakdown and abnormal loading patterns. Additionally, compensatory mechanisms can further strain surrounding muscles and joints, exacerbating pain. Our study investigated a potential solution: applying MEST, a treatment designed to stimulate muscle regeneration, to atrophied quadriceps muscles in animal models of knee OA. While MEST caused minor initial tissue trauma and inflammation, these gradually resolved as muscle restoration progressed over several weeks. Notably, the recovery of muscle weight, contractile force and hindlimb torque coincided with a significant decrease in OA pain. This evidence suggests that MEST presents a pioneering regenerative approach targeting skeletal muscle for managing OA pain.

One week after MEST treatment in MIA-treated animals, inserted cog filaments were found to induce muscle injury, as evidenced by increased levels of serum biomarkers [33] and a local inflammatory response (Figures 2I and 6B). While PDO filaments gradually degraded within the body over months [34], they appeared to stabilize with neighboring muscle tissue within weeks, resulting in minor and transient damage (Figures 2K and 6E). The specially designed cogs on these filaments [18] may restrict filament movement within the muscle to prevent excessive and prolonged damage. Interestingly, such changes in serum biomarkers and inflammation by MEST were not observed in the Naive animals even at M-W1 (Figures 2J and 6C), supporting that MEST causes minor damage that only significantly affects atrophied muscles in OA animals. When a muscle undergoes atrophy, its muscle fibers become smaller, denser and less resistant to stress [18, 35, 36], making it more susceptible to damage from inserted filaments and equipped cogs. The local increase in β-actin levels in normal muscle by MEST at M-W1 (Figure 2E) reflects the presence of a biochemical stimulus or injury [37, 38] but is not severe enough to trigger obvious pain, inflammation or changes in serum markers under normal condition (Figures 1B and F, 2J and 6C). The levels of local β-actin, which are present within muscle fibers, can be more sensitive than systemic serum biomarkers in detecting minor skeletal muscle injury.

Quadriceps weakness, a prevalent issue in knee OA patients, is associated with physical disability [39] and heightened knee loading rates [40], and is identified as a risk factor for knee OA development [14, 15]. While exercise is widely acknowledged as the most effective therapy for skeletal muscle atrophy so far [16, 41], it may not be suitable for all OA patients, especially elderly individuals who experience severe pain and functional limitations. In addition, despite considerable efforts to discover effective drug targets and chemical compounds to combat muscle loss [35, 36], successful pharmacological treatment options are still unavailable in clinical settings. A noteworthy finding in this regard is that intended but mild muscle injury caused by MEST in MIA-treated animals led to significant quadriceps recovery from atrophy at M-W3 (Figure 3). This outcome was consistent with a previous study showing that MEST restored atrophic rectus femoris muscle by increasing weight and fiber size as well as decreasing muscle density in OA animals at 5 and 9 weeks after MEST treatment [18]. Moreover, MEST effectively reversed impaired quadriceps contraction ability by increasing the maximum contraction and hindlimb torque force (Figure 3H and L; Supplementary Figure S2). The fundamental function of skeletal muscle is to generate contractile force, and decreased maximum isometric torque and electromyographic amplitude have been observed in OA patients [42]. Therefore, the successful restoration of impaired contraction ability could serve as a key indicator of enhanced quadriceps health achieved through MEST. The increase in muscle mass due to MEST treatment (Figure 3D) cannot be solely attributed to fibrosis since scar tissue lacks contractility and, therefore, should result in decreased muscle strength [43]. The fact that the cross-sectional area of individual muscle fibers also increased with MEST treatment further excludes this possibility [18]. Supporting these findings, a clinical trial confirmed a significant improvement in the maximum isometric contractile force of the affected leg in OA patients at 4 weeks after MEST treatment [20].

The recovery process of injured quadriceps in OA animals by MEST likely initiates with inflammatory responses. Particularly, macrophages are essential players in the regenerative capacity of injured skeletal muscle [23, 24]. These cells orchestrate the cleanup of damaged tissue and release a cocktail of cytokines and growth factors that promote the proliferation and differentiation of satellite cells [44, 45]. Additionally, macrophages may support the revascularization of the injured area, which is essential for the delivery of oxygen and nutrients to newly formed muscle cells [46]. The importance of macrophages in muscle repair is highlighted by the fact that depletion of these cells can lead to impaired regeneration [47]. The upregulation of CD68 expression (Figure 4), observed at both M-W1 and M-W3, signifies a sustained response of the body to tissue damage and the ongoing healing process in MIA-treated animals following MEST treatment [23, 24]. Macrophages present during tissue repair are not a single homogenous population but encompass a temporally regulated spectrum of activation states [48]. Notably, the dynamic changes in CD86 (Figure 4B and E) and CD206 (Figure 4H and K) expressions over weeks suggest a transition of the dominant type of macrophages from a pro-inflammatory M1 to an anti-inflammatory M2 activation state, which corresponds to the concurrent increase and resolution of IL-6 levels (Figure 6B and E). While M1 macrophages are typically dominant for 1–2 days and then replaced by M2 macrophages between 2 and 7 days post-muscle injury [49–51], since MEST-induced injury is repetitive and persistent during a certain period rather than a one-off event, the expression or dominance switch of macrophages may repeat and overlap, not strictly following the simple time course.

The early stage of muscle repair, involving the necrosis of damaged tissue and the initiation of an inflammatory response, is quickly succeeded by the activation of myogenic cells, or satellite cells. These cells then undergo proliferation, differentiation and fusion, according to the timely controlled specific signals emitted by the surrounding microenvironment, ultimately resulting in the formation of new myofibers and the restoration of a skeletal muscle [21, 22, 49, 51, 52]. It is noteworthy that MEST treatment to OA animals evoked notable changes in satellite cell dynamics. At M-W1, a significant increase in satellite cell number and activation, as evidenced by elevated PAX7 and MyoD expression (Figure 5B and H), indicated initiated muscle recovery, accompanied by higher levels of satellite cell activators such as IL-6 [53] and IGF-1 [54] (Figure 6B). As recovery progressed to M-W3, satellite cell activation remained high for several weeks; however, the lower activation rate compared to M-W1 (Figure 5E and K) and the return of activators to baseline levels (Figure 6E) implied a gradual winding down of the recovery process. The inconsistent changes observed among satellite cell activators in our study (Figure 6B) could be attributed to the immensely complex environment, characterized by spatiotemporally regulated cascades of direct and indirect cellular interactions [51]. Moreover, activators originate from diverse sources, including perturbed muscle tissue [55] and various cell types [56], further contributing to the variability in peak time points.

Our behavior data indicate that MEST treatment significantly reduces OA pain (Figure 1C and G), consistent with prior animal and clinical studies [18, 20]. The gradual pain relief over successive weeks fits well with the muscle recovery timeline in which the difference from the control group was significant at M-W3 (Figure 3). The variation in the initiation timing of pain relief reported in a prior study [18] might have resulted from disparities in the composition of control groups, as well as differences in the methodologies of behavioral experiments and the specific von Frey apparatus employed. Our findings reveal strong support in the extensive body of evidence showcasing the clinical benefits of exercise among OA patients [57, 58]. Considering the substantial influence of muscle weakness on both pain and functionality in OA [12–15, 39, 40], muscle strengthening stands as a pivotal element in the majority of knee OA exercise regimens [16, 41]. Therefore, it is plausible that the restored quadriceps muscles resulting from MEST treatment contribute to the protection and stabilization of the knee joint [30], the absorption of shock [31, 32], and the preservation of optimal joint mechanics [59]. These combined effects are likely to alleviate OA knee pain by reducing stress on the joint and preventing further damage, resulting in the alleviation of peripheral sensitization [19], even in the absence of improvement in joint structure [18]. Enhanced quadriceps muscle strength correlates with reduced pain, diminished challenges in accomplishing daily activities and improved mobility among individuals with knee OA [29]. Moreover, the alleviation of pain facilitates increased movement, which reciprocally fortifies the quadriceps, establishing a positive feedback loop in pain relief.

The current landscape of OA treatments, encompassing medication, physical therapy, joint replacements, and lubrication injections, reveals significant limitations, providing only partial pain relief to a subset of patients and often accompanied by adverse effects [1, 2]. This underscores the pressing demand for novel therapeutic strategies. In this context, MEST represents a promising alternative treatment option, offering significant efficacy and a favorable safety profile by uniquely focusing on strengthening skeletal muscles rather than directly targeting the affected joint. This complementary approach holds the potential for seamless integration with existing treatments, thereby potentially enhancing therapeutic outcomes for individuals affected by OA.

## Conclusion

Collectively, our results unveiled that the MEST intervention in atrophic quadriceps muscles of OA animals induced minor muscle damage, subsequently activating both macrophages and satellite cells in concurrence with the upregulation of their activators. These changes exhibited a gradual attenuation over several weeks, ultimately contributing to muscle recovery from atrophy and relief from OA pain. Our findings provide strong support for the notion that targeting the restoration of key skeletal muscles associated with the OA joint through MEST presents a promising avenue for new OA pain treatment, although it may not be a direct remedy for OA itself. Further research is needed to investigate the effects of MEST on the knee joint to deepen our understanding of the mechanisms underlying OA pain relief. As MEST represents a novel approach, further rigorous clinical trials are also crucial to establish its safety and efficacy.

## Supplementary Material

rbae077_Supplementary_Data

## Data Availability

The data that support the findings of this study are available in the results and/or supplementary material of this article.
