# Supplementary material for: Quadriceps recovery and pain relief in knee osteoarthritis rats by cog polydioxanone filament insertion
Source: Regen Biomater. 2024 Jun 21;11:rbae077. doi: 10.1093/rb/rbae077 (PMC11226885; doi:10.1093/rb/rbae077)
Supplement: rbae077_Supplementary_Data [file rbae077_supplementary_data.pdf]

Supplementary Table 1. List of antibodies for immunofluorescence and western blot.

Antibody	Host	Manufacturer	Cat. No.	Dilution
Primary antibody				
CD68	Rabbit	Abcam	ab125212	1:1000
CD86	Rabbit	Novusbio	NBP2-67417	1:500
CD206	Rabbit	Cell signaling technology	#24595	1:1000
Laminin	Rabbit	Novusbio	NB300-144	1:1000
Pax7	Mouse	Novusbio	NBP2-32894	1:1000
MyoD	Mouse	Invitrogen	MA1-41017	1:500
IL-6	Mouse	ABcam	ab9324	1:1000
TNF-α	Rabbit	ABcam	ab66579	1:1000
IGF-1	Rabbit	Alomone labs	ANT-046	1:1000
TGFβ-1	Mouse	Invitrogen	MA5-16949	1:1000
β-actin	Mouse	Cell Signaling Technology	3700S	1:10000
GAPDH	Rabbit	ABFrontier	LF-PA0018	1:10000
Secondary antibody				
Alexa Fluor® 488 AffiniPure F(ab')$_2$ Fragment Donkey Anti-Rabbit IgG (H+L)	Donkey	Jackson ImmunoResearch	711-546-152	1:1000
Cy™3 AffiniPure F(ab')$_2$ Fragment Donkey Anti-Mouse IgG (H+L)	Donkey	Jackson ImmunoResearch	715-166-151	1:1000
Anti-mouse IgG, HRP linked antibody	Horse	Cell Signaling Technology	7076S	1:5000
Anti-rabbit IgG, HRP linked antibody	Goat	Cell Signaling Technology	7074S	1:5000

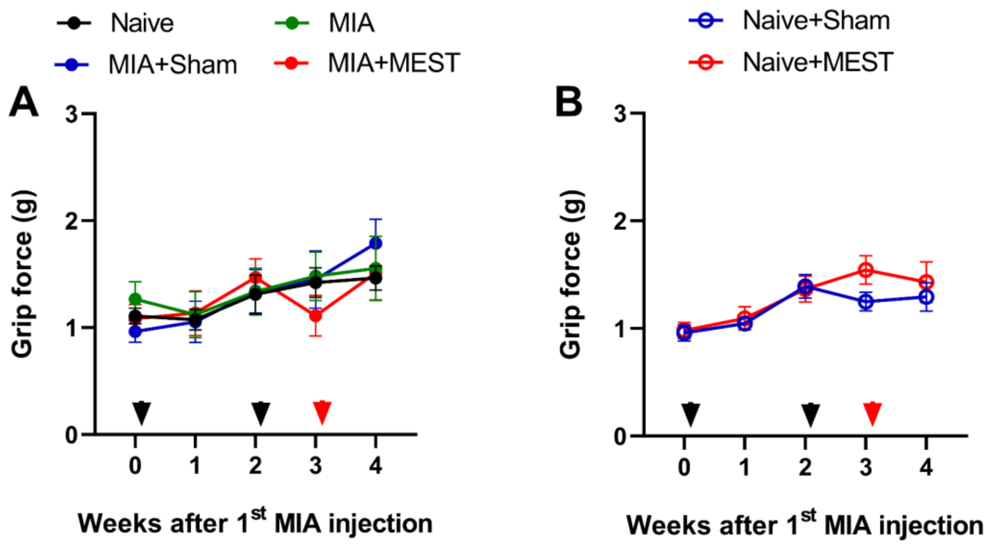

Supplementary Figure 1. MIA or MEST treatment does not affect the grip force of hindpaws. (A and B) Changes in hindpaw grip force over 4 weeks. n=11-12 per group. Data are presented as mean ± SEM. The data were analyzed using two-way repeated measures ANOVA followed by Tukey's (A) or Sidak's (B) test.

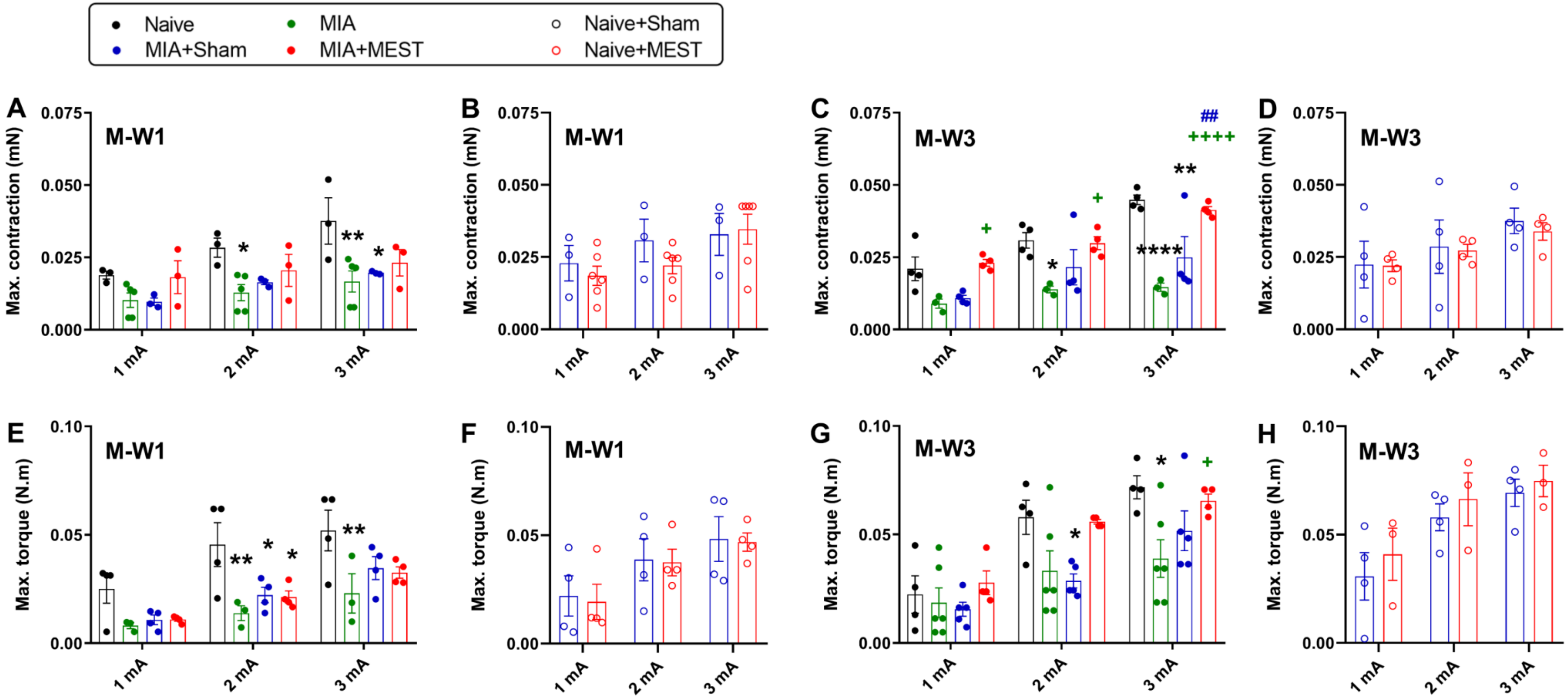

Supplementary Figure 2. MEST restores quadriceps function. (A-D) Recovery of contraction force. The maximal contraction force and hindlimb torque force were measured to evaluate quadriceps contraction ability. The maximal contraction force of the quadriceps elicited by electrical stimulation at M-W1 (A and B) and M-W3 (C and D). n=3-6 per group. (J-M) Recovery of torque force of the hindlimb. The maximal torque force of the hindlimb elicited by electrical stimulation of quadriceps at M-W1 (E and F) and M-W3 (G and H). n=3-6 per group. Electrical stimulation with 10-ms pulse durations at amplitudes of 1, 2, or 3 mA was delivered to elicit quadriceps contraction and hindlimb torque. * $p<0.05$, ** $p<0.01$, **** $p<0.0001$ vs. Naïve. + $p<0.05$, ++++ $p<0.0001$ vs. MIA. ## $p<0.01$ vs. MIA+Sham. Data are presented as mean ± SEM. The data were analyzed using two-way repeated measures ANOVA followed by Tukey's (A, C, E, G) or Sidak's (B, D, F, H) test.

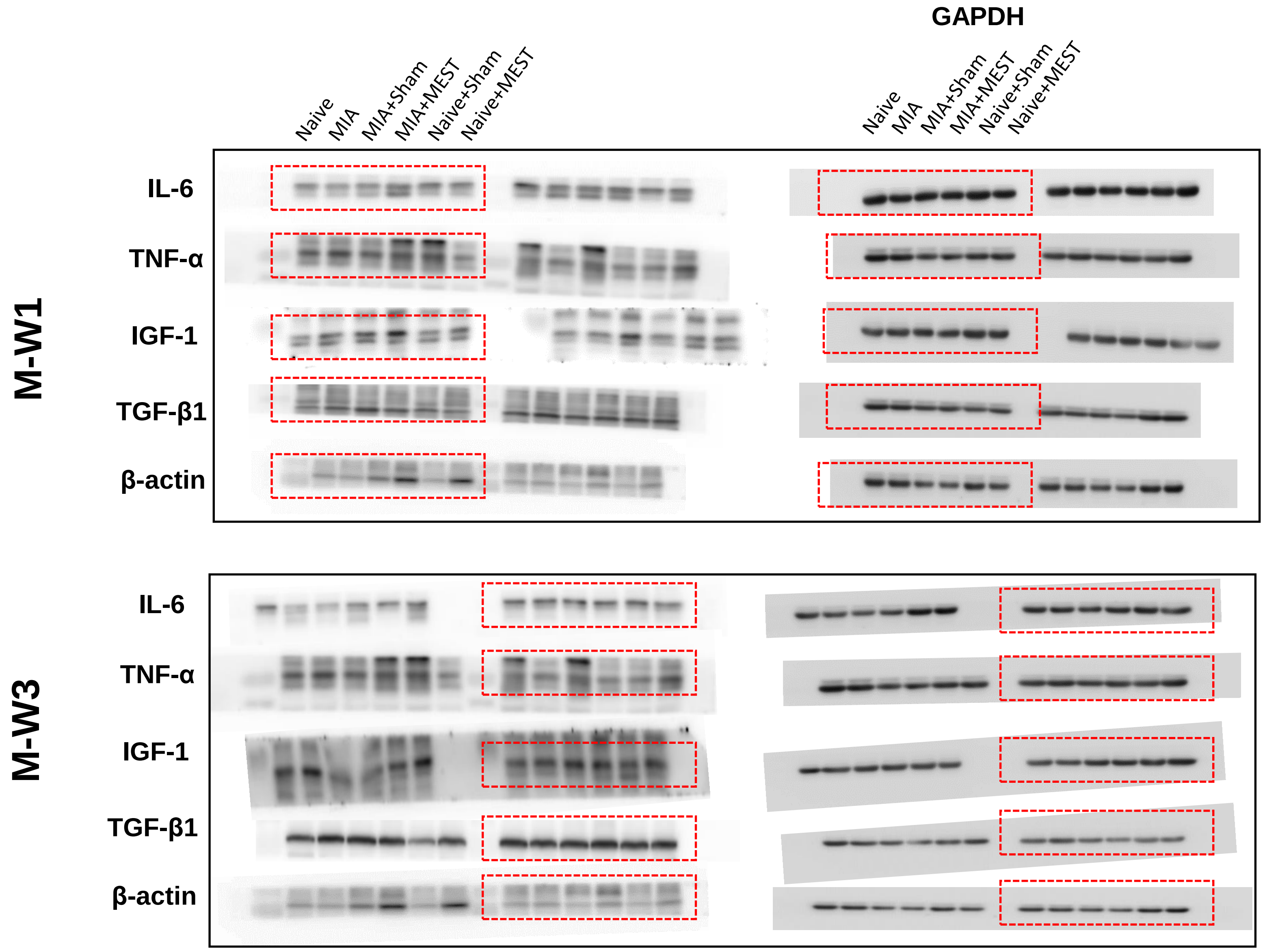

Supplementary Figure 3. Original uncropped western blot data for presented blots in Figures 2 and 6. The red dotted squares indicate the blots used in the figures. The lower and upper portions of each membrane were cut in accordance with the ladder guide after protein transfer and before exposure.
